# Effects of β-glucan Rich Barley Flour on Glucose and Lipid Metabolism in the Ileum, Liver, and Adipose Tissues of High-Fat Diet Induced-Obesity Model Male Mice Analyzed by DNA Microarray

**DOI:** 10.3390/nu12113546

**Published:** 2020-11-19

**Authors:** Kento Mio, Chiemi Yamanaka, Tsubasa Matsuoka, Toshiki Kobayashi, Seiichiro Aoe

**Affiliations:** 1 Studies in Human Life Sciences, Graduate School of Studies in Human Culture, Otsuma Women’s University, Chiyoda-ku, Tokyo 102-8357, Japan; mio.kento@hakubaku.co.jp; 2 Research and Development Department, Hakubaku Co. Ltd., Chuo-City, Yamanashi 409-3843, Japan; matsuoka.tsubasa@hakubaku.co.jp (T.M.); k.toshiki@hakubaku.co.jp (T.K.); 3 The Institute of Human Culture Studies, Otsuma Women’s University Chiyoda-ku, Tokyo 102-8357, Japan; chiemiy@gmail.com

**Keywords:** barley, β-glucan, dietary fiber, microarray, short chain fatty acids, lipid metabolism.

## Abstract

We evaluated whether intake of β-glucan-rich barley flour affects expression levels of genes related to glucose and lipid metabolism in the ileum, liver, and adipose tissues of mice fed a high-fat diet. C57BL/6J male mice were fed a high-fat diet supplemented with high β-glucan barley, for 92 days. We measured the expression levels of genes involved in glucose and lipid metabolism in the ileum, liver, and adipose tissues using DNA microarray and q-PCR. The concentration of short-chain fatty acids (SCFAs) in the cecum was analyzed by GC/MS. The metabolic syndrome indices were improved by barley flour intake. Microarray analysis showed that the expression of genes related to steroid synthesis was consistently decreased in the liver and adipose tissues. The expression of genes involved in glucose metabolism did not change in these organs. In liver, a negative correlation was showed between some SCFAs and the expression levels of mRNA related to lipid synthesis and degradation. Barley flour affects lipid metabolism at the gene expression levels in both liver and adipose tissues. We suggest that SCFAs are associated with changes in the expression levels of genes related to lipid metabolism in the liver and adipose tissues, which affect lipid accumulation.

## 1. Introduction

Metabolic syndrome is widely known as a multi-factorial disorder with symptoms such as hypertension, hyperglycemia, insulin resistance, and visceral fat obesity. This syndrome is promoted by irregular eating, lack of exercise, excessive drinking of alcohol and stress, which may cause risk factors such as cardiovascular disease, coronary disease, and type 2 diabetes [1]. In particular, quality of diet is closely associated with the development of metabolic syndrome. In recent years, the beneficial effects of whole-grains have become apparent: a meta-analysis of cohort studies showed that a high consumption of whole-grains is associated with reduced incidence of type 2 diabetes [2]. Another report showed that whole-grain consumption (48–80 g/3–5 serving/day) reduced the incidence of obesity, type 2 diabetes, and concentrations of total and LDL-cholesterol in serum [3]. A systematic review also reported that intake of a whole-grain and fiber diet reduced the risk of type 2 diabetes, the incidence of being overweight and obesity in Japanese men and women [4]. Whole-grain and grains are good sources of fiber, and the effect of dietary fiber on lipid metabolism has been studied over a long period [5,6,7].

Barley, a cereal grain, contains higher amounts of β-glucan compared with other grains such as oats, rye, and wheat. Since barley β-glucan is distributed throughout the kernel, the amount of β-glucan in refined barley is unchanged. Barley β-glucan is a polysaccharide polymer in which glucose is linked by β-glycoside bonds (β-1,3–1,4), and is almost soluble in water [8]. β-Glucan increases water solubilization in the stomach and small intestine and delays the absorption of other nutrients [9]. These effects suppress increases in postprandial blood glucose, and maintains satiety, thereby improving insulin resistance and suppressing visceral fat accumulation by reduced excessive insulin secretion [10]. It has also been reported that blood cholesterol levels were reduced by barley intake in vivo and human studies [11,12,13]. Studies have indicated that the mechanism of barley β-glucan involves inhibition of cholesterol synthesis in the liver, inhibition of bile acid reabsorption and alteration of cholesterol metabolism due to decreased insulin secretion and promotion of cholesterol excretion [14,15].

Water-soluble dietary fibers such as barley β-glucan are fermented by bacteria in the cecum to the distal colon. Fermentation produces short-chain fatty acids (SCFAs) such as acetic acid, propionic acid, and butyric acid as metabolites which serve as energy sources for the host. It was recently reported that SCFAs affect lipid metabolism in several organs such as liver, muscle, and brown adipose tissue [16]. Acetic acid is mainly metabolized in the liver and is involved in fat and cholesterol synthesis in peripheral tissues [17]. Propionic acid is mainly metabolized in the liver and intestinal tract, and butyric acid is involved in cell differentiation and maintenance of intestinal barrier formation in the intestinal tract [18]. These SCFAs also contribute to reducing intestinal pH, which inhibits the absorption of toxic products such as *p*-cresol and phenols [19]. Moreover, recent studies have indicated the SCFAs act in the host metabolism as signal molecules, regulating intestinal hormone secretion and insulin signaling via SCFA receptors [20].

The findings of the above studies support the beneficial effects of barley β-glucan in normalizing cholesterol concentration, improving glucose tolerance, and reducing visceral fat. These effects are considered to be related to the physiological functions of delaying digestion and absorption of nutrients in the diet from the digestive tract, and physiological functions of host energy by gut microbiota-derived SCFAs. However, the mechanism of barley β-glucan affecting lipid metabolism is still not understood at the gene level. There are very few reports which confirm the effects of barley β-glucan on lipid metabolism in several organs, such as intestinal tract, adipose, and liver tissues; therefore, it is still debatable whether the intake of β-glucan-rich barley flour is involved in lipid metabolism at the gene level.

To investigate the effect of β-glucan-rich barley on glucose and lipid metabolism, we measured gene expression levels using DNA microarrays, which are a tool to comprehensively detect total gene expression. In recent years, several studies have clarified the physiological effects of dietary fiber materials using DNA microarrays. Drew et al. reported that genes involved in the intestinal barrier function and metabolism of the intestinal epithelium were upregulated in the cecum of mice fed inulin or β-glucan extract of barley [21]. Other studies have reported that the expression of genes involved in fatty acid oxidation and lipid transport in skeletal muscle are upregulated in mice fed psyllium [22]. The purpose of this study was to investigate whether the intake of β-glucan-rich barley flour affects the expression levels of genes related to glucose and lipid metabolism in the ileum, liver, and adipose tissues of mice fed a high-fat diet using a DNA microarray and subsequent analysis by q-PCR. We also investigated the relationship between gene expression levels in each organ and SCFAs in the cecum.

## 2. Materials and Methods

### 2.1. Sample Preparation and Chemical Analysis

A new hulless barley cultivar, “Beau Fiber” (BF), was used in our experiments. BF flour pearled to 70% yield was obtained from the National Agriculture and Food Research Organization (Tsukuba, Japan). Total dietary fiber was analyzed using the AOAC 991.43 method [23]. β-Glucan content was analyzed using the McCleary method (AOAC 995.16) [24]. Protein and lipid content in BF were analyzed by the Kjeldahl and acid hydrolysis method, respectively. The nutritional content of BF is shown in Table 1.

### 2.2. Animals and Study Design

Four-week-old male C57BL/6J mice were purchased from Charles River Laboratories Japan, Inc. (Yokohama, Japan). Mice were housed on a 12 h light/dark cycle (light on at 07:30 h) under the conditions of constant air exchange at a temperature of 22 ± 1 °C and humidity of 50 ± 5%. Mice were acclimatized for one week on commercial chow (NMF, Oriental Yeast Co., Ltd., Shiga, Japan), before they were randomly assigned into two groups according to body weight (*n* = 10 per group). Mice were individually housed in plastic cages, and given the experimental powdered diets (Table 2). A high fat diet was prepared by the addition of lard to the AIN-93G diet (fat energy ratio 50%). The control and BF diets were supplemented with cellulose and BF flour, respectively, to give 5% total dietary fiber (Table 1). Mice were given free access to water and experimental diets during the whole experimental period of 92 days. Food intake and body weights were monitored three times a week during the study. Oral glucose tolerance test (OGTT) were performed in mice after 8 h fasting during the 11th week of the experimental diets. Mice were orally administered 20% glucose (1.5 g/kg body) and blood samples were collected from the tail at 0, 15, 30, 60, 120 min, before glucose levels were analyzed using electrode method (Glutest Neo Super, Sanwa Kagaku Kenkyusho Co., Ltd., Aichi, Japan). At the end of the study, mice were fasted for 8 h and sacrificed by isoflurane/CO_2_ anesthesia. Blood samples were then collected from the postcaval vein, centrifuged to obtain serum which was stored at −80 °C until biochemical analysis. The weights of liver, cecum, and adipose (epididymal fat, retroperitoneal fat, mesenteric fat) tissues were measured and then ileum, liver, and adipose (epididymal fat) tissues were immediately soaked in RNA protect Tissue Reagent (RNAlater, Qiagen, Hilden, Germany), while samples of liver and cecum contents were stored at −30 °C. The animal protocol was approved by the Animal Research Committee of Otsuma Women’s University (Tokyo, Japan) and was implemented in accordance with their regulations (No. 17012, February 2018).

### 2.3. Concentration of Liver Lipid and Biochemical Analysis in Serum

Liver lipid concentration was measured using Folch’s method [25]. Chloroform-methanol solution (2:1 *v*/*v*) was used to extract lipids from the liver, and isopropanol containing 10% polyoxyethylene octylphenyl ether (Triton X-100, FUJIFILM Wako Pure Chemical Corporation, Osaka, Japan) was added to dissolve the lipids. Triglyceride and cholesterol concentrations in the extracts were measured enzymatically using the Cholesterol E-test and Triglyceride E-test, respectively (FUJIFILM Wako Pure Chemical Corporation, Osaka, Japan).

Total cholesterol (TC), low-density lipoprotein (LDL), high-density lipoprotein (HDL)-cholesterol, triglycerides (TG) and non-esterified fatty acids (NEFA) were measured in mice serum using Hitachi 7180 auto-analyzers at the Nagahama Research Institute (Oriental Yeast Co., Ltd., Shiga, Japan). Enzyme-linked immunosorbent assays (ELISAs) were used to measure serum insulin (mouse insulin ELISA kit, Shibayagi Co., Ltd., Gunma, Japan) and leptin (mouse leptin immunoassay kit R & D Systems, Inc., Minneapolis, MN, USA) concentrations.

### 2.4. Short-Chain Fatty Acids Analysis in Cecum Contents

SCFA concentration in the cecum contents was analyzed by gas chromatography-mass spectrometry (GC/MS) based on a previous report [26]. Twenty mg of cecum contents was added to a 2 mL microtube containing 100 μL of internal standard (100 μM crotonic acid), 50 μL of HCl, 300 μL of diethyl ether, and 5 mm stainless beads (AS ONE Corp., Osaka, Japan), and homogenized using a Tissue Lyser II (Qiagen, Hilden, Germany) at 2000 rpm for 2 min twice. After homogenates were centrifuged (3000 rpm, at 25 °C, for 15 min), 80 μL of supernatant (ether layer) was collected into a glass vial (Agilent Technologies Japan, Ltd., Tokyo, Japan), and mixed with 16 μL of *N*-tert-butyldimethylsilyl-*N*-methyltrifluoroacetamide (derivatization reagent). Vials were then sealed with a cap, and heated at 80 °C for 20 min. Vials were then placed at room temperature for 48 h for derivatization. The derivatized samples were analyzed using the 7890B GC system equipped with a 5977A mass selective detector (Agilent Technologies Japan, Ltd., Tokyo, Japan) and DB-5MS column (30 m × 0.53 mm). The oven temperature was initially kept at 60 °C, then ramped up to 120 °C at a rate of 5 °C/min. The oven was then ramped to 300 °C at a rate of 20 °C/min, and finally maintained at 300 °C for 2 min. Helium was used as the carrier gas at 1.2 mL/min. The temperature of the front inlet, transfer line, and electron impact ion source were set at 250, 260, and 230 °C, respectively. The mass spectral data were collected in a selective ion monitoring mode. Concentrations of SCFA were calculated by comparing the peak area with the internal standard.

### 2.5. DNA Microarray Analysis

The RNeasy Mini kit (Qiagen, Hilden, Germany) was used to extract total RNA in ileum, liver, and adipose (epididymal fat) tissues. The RNA integrity number (RIN) of all total RNA samples was checked by a visual inspection of the Bioanalyzer electropherograms (Agilent technologies Japan, Ltd., Tokyo, Japan). Based on a previous report [27], samples with RIN values higher than 6.5 were used for the analysis. Each RNA was mixed equally to obtain pooled RNA for each group (n = 10, per group). Total RNA (100 ng) was processed for use on the microarray using the GeneChip WT PLUS Reagent Kit (Thermo Fisher Scientific, Inc., Waltham, MA, USA) according to the manufacturer’s instructions. The resultant single-strand cDNA was fragmented and labeled with biotin, then hybridized to the GeneArray Mouse 2.0ST Array. The arrays were washed, stained and scanned using the Affymetrix 450 Fluidics Station and GeneChip Scanner 3000 7G (Thermo Fisher Scientific, Inc., Waltham, MA, USA) according to the manufacturer’s recommendations (Thermo Fisher Scientific, Inc., Waltham, MA, USA). Expression values were generated using Expression Console software, version 1.3 (Thermo Fisher Scientific, Inc.) with default robust multichip analysis parameters. These analyzes were performed by Kurabo Industries Ltd. (Osaka, Japan)

Raw and standardized (logarithmic transformation) microarray data were registered in Gene Expression Omnibus (GEO) at the National Center for Biotechnology Information (NCBI). GEO accession number was GSE157828.

### 2.6. mRNAs Expression Analysis in Ileum, Liver, and Adipose Tissues

mRNA expression levels were analyzed by Applied Biosystems Quant3 Real-Time polymerase chain reaction (PCR) system. Synthesized cDNA was used to measure the mRNA expression level using the 2^−∆∆CT^ method and Power-up SYBR^®®^ Green PCR Master Mix (Thermo Fisher Scientific, Waltham, MA, USA). We used threshold cycle (CT) for data analysis, which indicates the fractional cycle number as the amount of amplified target reaches a fixed threshold. The ΔCT is the difference in threshold cycles for target genes compared to the reference gene, 36B4. The ∆∆CT is the difference between the ∆CT for the control group and the ∆CT for the BF group. Relative expression levels are showed as fold changes to the control group (arbitrary unit). Primer sequences are shown in Table S1.

### 2.7. Statistical Analysis

All statistical analyses were performed using R software (ver. 3.6.3, R Foundation for Statistical Computing, Vienna, Austria). Data are presented as mean ± standard deviation (SD) of the mean. Significant differences between the control group and BF group were analyzed by Student’s *t*-test if homoscedasticity and normality were confirmed, and by Wilcoxon test if not confirmed. Difference were assessed with two-side test with an α level of 0.05. The relationships among the expression levels of mRNA related to lipid metabolism and SCFAs were assessed using Spearman’s rank correlation coefficient.

## 3. Results

### 3.1. Changes in Body Weight, Food Intake, and Organ Weights

Body weight, food intake, and organ weights in mice fed the experimental diets are shown in Table 3. There were no significant differences in final weight, body weight gain, food intake, and food efficiency ratio between the two experimental groups. Organ weights in mice fed the experimental diets are shown in Table 4. Liver weight and the weights of retroperitoneal and mesenteric fat were significantly lower in the BF group compared with the control group (*p* < 0.05). The weight of cecum with digesta was significantly higher in the BF group compared with the control group (*p* < 0.05).

### 3.2. Concentrations of the Liver and Serum Lipid

The concentration of liver lipids, and serum biochemical markers are shown in Table 5. Accumulation of liver cholesterol and triglyceride was significantly lower in the BF group compared with the control group (*p* < 0.05). Serum TC, LDL and leptin concentrations were significantly lower in the BF group compared with the control group (*p* < 0.05). Serum TG concentration was significantly higher in the BF group compared with the control group (*p* < 0.05). There were no significant differences in serum HDL, NEFA, and insulin concentrations between the experimental groups.

### 3.3. Oral Glucose Tolerance Test (OGTT)

**Figure 1:**
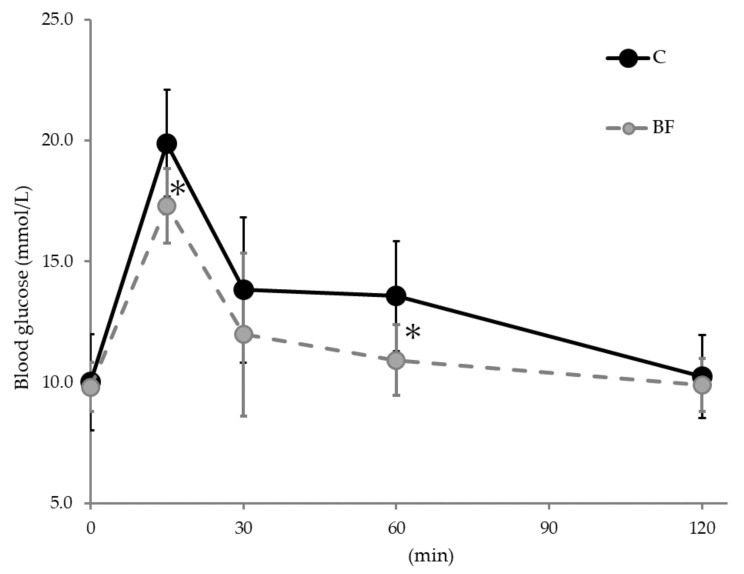
Blood glucose levels in the oral glucose tolerance test (OGTT). Data are shown as mean ± SD. * Significantly different from the control group (*p* < 0.05). C: Control group, BF: Beau fiber group.

The OGTT results are shown in Figure 1. Blood glucose levels after glucose administration were significantly lower in the BF group compared with the control group at 15 and 60 min (*p* < 0.05). No significant differences were observed at other times.

### 3.4. Concentrations of SCFAs in Cecum Contents

Concentrations of SCFAs and organic acids in the cecum are shown in Table 6. The amounts of acetic acid, propionic acid, lactic acid, succinic acid, and total SCFAs were higher in the BF group compared with the control group (*p* < 0.05). No significant difference in the amount of butyric acid was observed between the experimental groups.

### 3.5. Gene Expression Profiles of the Ileum, Liver, and Adipose Tissues from Microarray

#### 3.5.1. Differential Expressed Genes (DEGs) by Microarray Analysis

The microarray analyses of each organ was compared by logarithmically converting values (Log-Ratio) of gene expression levels between the experimental groups, excluding non-coding genes. In the ileum of mice in the BF group, the expression levels of 1536 genes had increased >1.3 fold and 1529 genes had decreased <0.77 fold when compared with the control group (Table S2). In the liver of mice in the BF group, the expression levels of 2197 genes had increased >1.3 fold and 2054 genes had decreased <0.77 fold when compared with the control group (Table S3). In the adipose tissue of mice in the BF group, the expression levels of 2111 genes had increased >1.3 fold and 1878 genes had decreased <0.77 fold when compared with the control group (Table S4). The DEGs were annotated with gene symbols using Transcriptome Viewer (Kurabo Industries Ltd., Osaka, Japan) and subjected to further analyses.

#### 3.5.2. DEG Profiles Related to Lipid Metabolism

**Figure 2:**
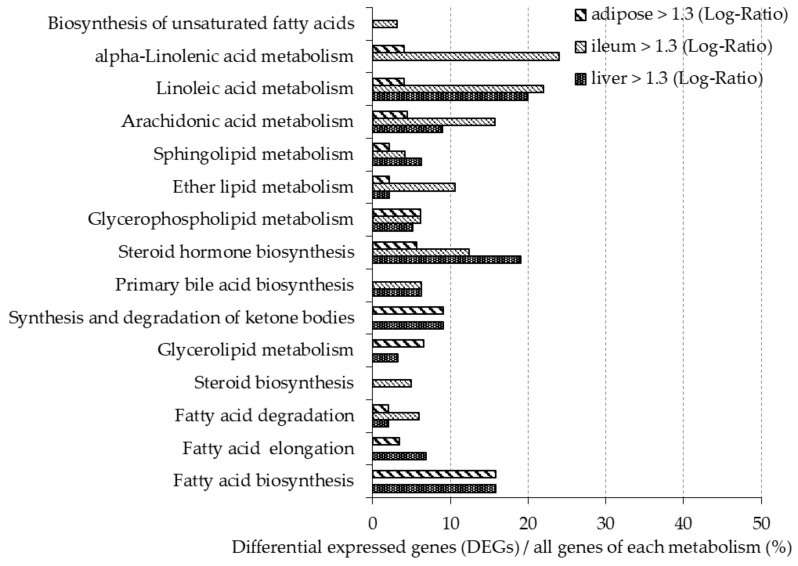
In mice fed the BF diet, the ratio of Differential Expressed Genes (DEGs) upregulated (gene expression > 1.3-fold difference compared with the control group) in each organ is shown. The *y*-axis shows the lipid metabolic pathway described in Kyoto Encyclopedia of Gene and Genome (KEGG). The *x*-axis shows the percentage of the DEGs of all genes involved in each metabolic pathway.

**Figure 3:**
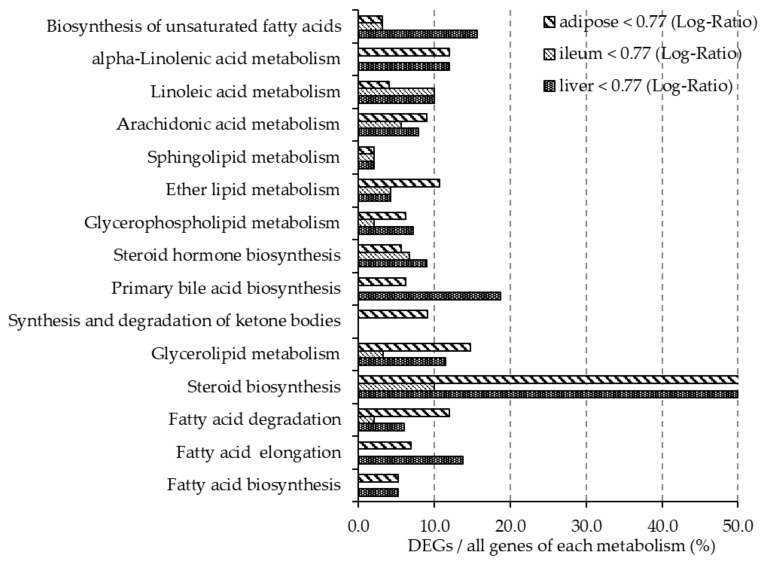
In mice fed the BF diet, the ratio of DEGs downregulated (gene expression < 0.77-fold difference compared with the control group) in each organ is shown. The *y*-axis shows the lipid metabolic pathways described in KEGG. The *x*-axis shows the percentage of DEGs of all genes involved in each metabolic pathway.

Figure 2 and Figure 3 show the ratio of DEGs to all genes involved in the lipid metabolism pathway, as described in the Kyoto Encyclopedia of Gene and Genome (KEGG) Pathway Database [28]. More than 10% of genes involved in steroid biosynthesis (mmu00140) were downregulated in all organ tissues. In the liver, more than 10% of genes involved in fatty acid elongation (mmu00062), glycerolipid metabolism (mmu00561), primary bile acid biosynthesis (mmu00120), biosynthesis of unsaturated fatty acids (mmu01040), and alpha-linolenic acid metabolism (mmu00592) were downregulated, while more than 10% of genes involved in steroid hormone biosynthesis (mmu00140), linoleic acid metabolism (mmu00591), and fatty acid biosynthesis (mmu00061) were upregulated. In adipose tissue, more than 10% of genes involved in fatty acid degradation (mmu00071), glycerolipid metabolism, ether lipid metabolism (mmu00565), and alpha-linolenic acid metabolism were downregulated, while more than 10% genes of genes involved in fatty acid biosynthesis were upregulated. In the ileum, more than 10% of genes involved in steroid hormone biosynthesis, ether lipid metabolism, arachidonic acid metabolism (mmu00590), linoleic acid metabolism, and alpha-linolenic acid metabolism were upregulated.

#### 3.5.3. DEGs Profiles Related to Carbohydrate Metabolism

**Figure 4:**
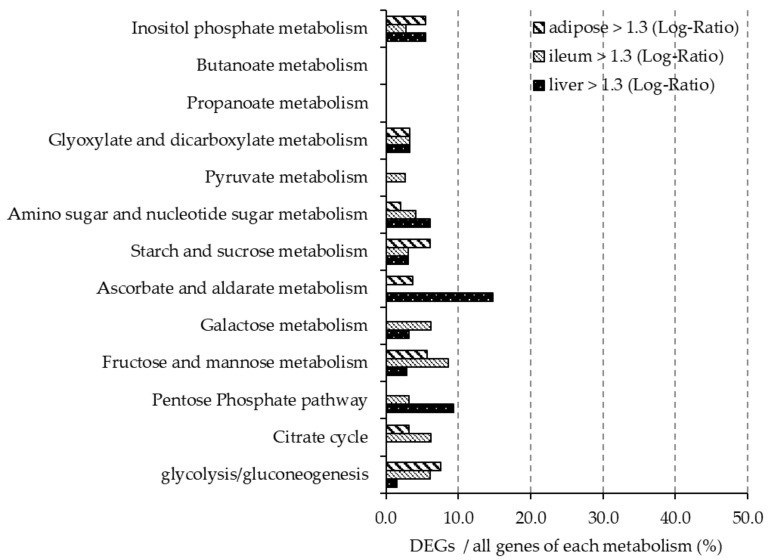
In mice fed the BF diet, the ratio of DEGs upregulated (gene expression > 1.3-fold difference compared with the control group) in each organ is shown. The *y*-axis shows the carbohydrate metabolic pathway described in KEGG. The *x*-axis shows the percentage of DEGs of all genes involved in each metabolic pathway.

**Figure 5:**
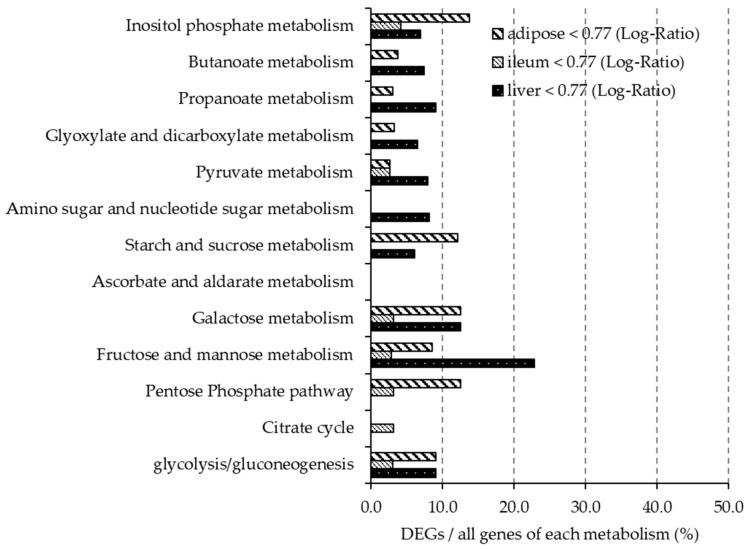
In mice fed the BF diet, the ratio of DEGs downregulated (gene expression < 0.77-fold difference compared with the control group) in each organ is shown. The *y*-axis shows the carbohydrate metabolic pathway described in KEGG. The *x*-axis shows the percentage of DEGs of all genes involved in each metabolic pathway.

Figure 4 and Figure 5 show the ratio of DEGs to all genes involved each carbohydrate metabolism pathway, as described in the KEGG Pathway Database. In the liver, more than 10% of genes involved in ascorbate and aldarate metabolism (mmu00053) were upregulated, and more than 10% of genes involved in galactose metabolism (mmu00052) and fructose and mannose metabolism (mmu00051) were downregulated. Less than 10% of the genes involved in carbohydrate pathways in the ileum were differentially expressed. In adipose tissue, more than 10% of the genes involved in inositol phosphate metabolism, starch and sucrose metabolism (mmu00500), galactose metabolism and pentose phosphate metabolism (mmu00040) were downregulated.

### 3.6. mRNA Expression Levels in Ileum, Liver, and Adipose Tissues Using q-PCR

**Figure 6:**
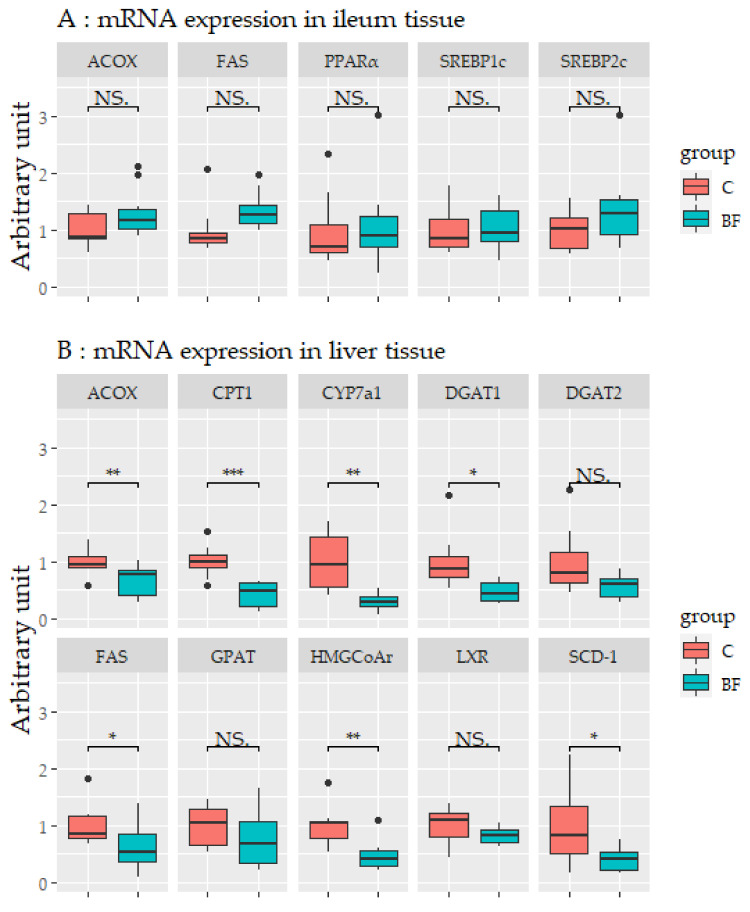

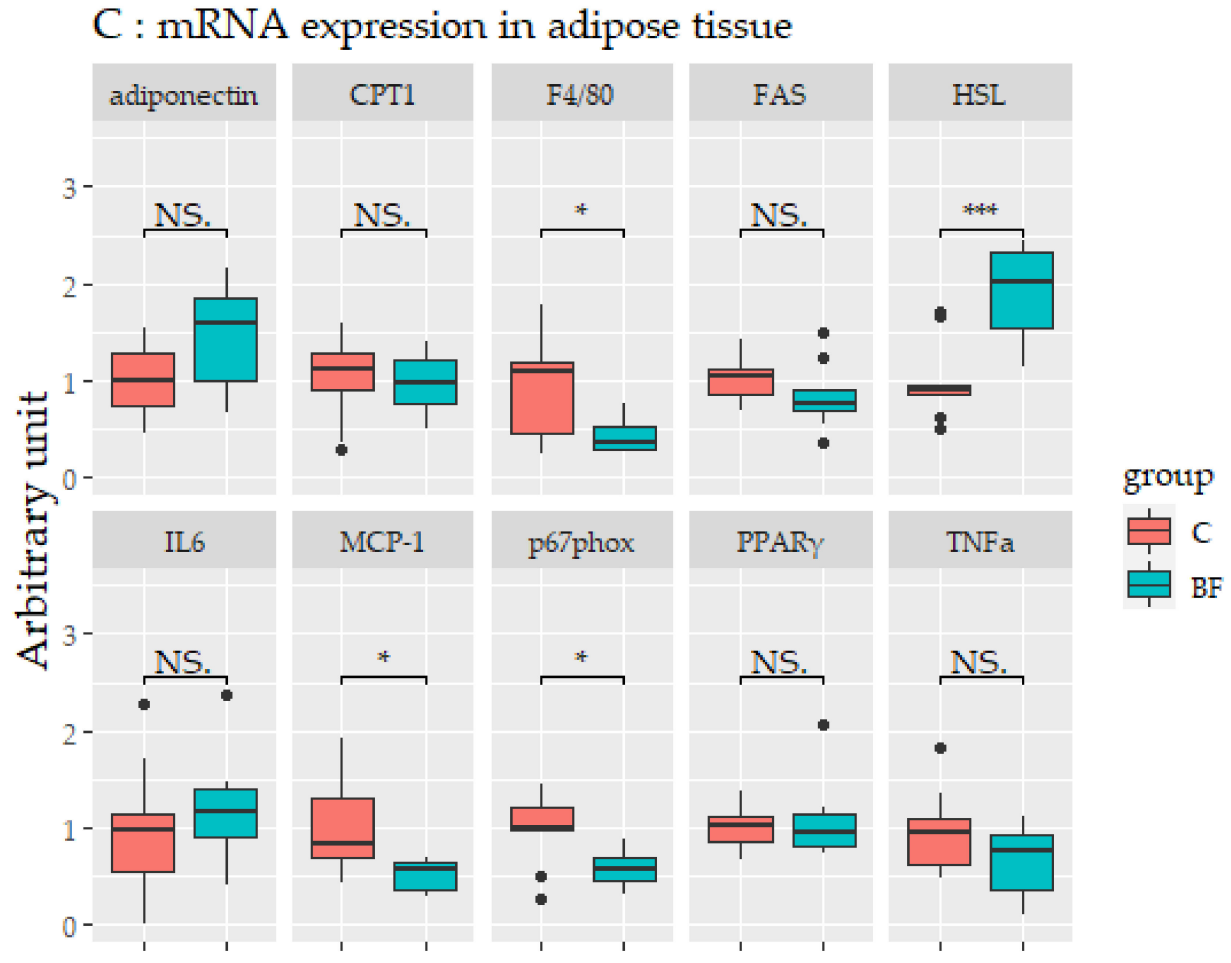
The expression levels of mRNA in the ileum (**A**), liver (**B**), and adipose (**C**) tissues. Values are means ± SD. * *p* < 0.05, ** *p* < 0.01, *** *p* < 0.001 significantly different from the control group. NS.: not significant. ACOX, Acyl-CoA oxidase; FAS, Fatty acid synthase; PPARα, Peroxisome proliferator-activated receptor-α; SREBP1c, Sterol regulatory element-binding protein-1c; SREBP2, Sterol regulatory element-binding protein-2; CPT1, Carnitine palmitoyl transferase 1; CYP7a1, Cholesterol 7alpha-hydroxylase 1; DGAT1, Diacylglycerol acyltransferase-1; DGAT2, Diacylglycerol acyltransferase-2; GPAT, glycerol-3-phosphate acyltransferase; HMGCoAr, Hydroxymethylglutaryl-CoA reductase, reductase; LXR, Liver X receptor; SCD-1, Stearoyl-CoA desaturase 1; HSL, Hormone-Sensitive Lipase; IL6, Interleukin-6; MCP-1, Monocyte chemotactic protein-1; p67phox, NADPH oxidase subunit p67 phox; PPARγ, Peroxisome proliferator-activated receptor-γ; TNF-α, Tumor necrosis factor-α. C: Control group, BF: Beau fiber group.

mRNA expression levels in the ileum, liver, and adipose tissues are shown in Figure 6. No significant differences in mRNA expression levels were observed in the ileum (Figure 6A). In liver, the mRNA expression levels of fatty acid synthase (FAS), acyl-CoA oxidase (ACOX), diacylglycerol o-acyltransferase-1 (DGAT1), stearoyl-CoA-desaturase-1 (SCD-1), carnitine palmitoyltransferase-1 (CPT-1), 3-hydroxy-3-methyl-glutaryl-coenzyme A reductase (HMG-CoA r), and cytochrome P450 7A1 (CYP7a1) were significantly lower in the BF group compared with the control group (*p* < 0.05) (Figure 6B). In adipose tissue, the mRNA expression levels of hormone-sensitive lipase (HSL) was higher in the BF group compared with the control group (*p* < 0.05), and the mRNA expression of monocyte chemotactic protein 1 (MCP-1), F4/80, and NADPH oxidase subunit p67 phox (p67^phox^) were significantly lower in the BF group compared with the control group (*p* < 0.05) (Figure 6C).

### 3.7. Correlation Analysis between SCFAs and Expression of mRNA Related to Lipid Metabolism in Liver and Adipose Tissues

**Figure 7:**
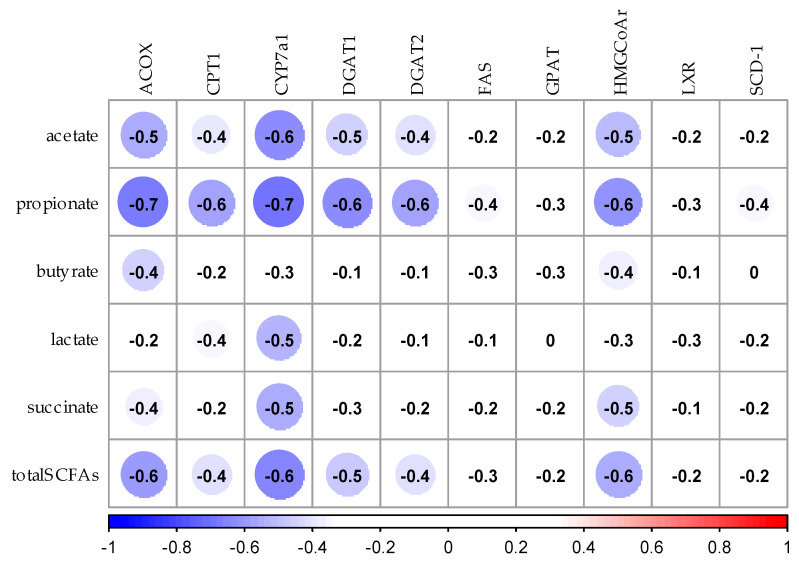
Spearman’s rank correlation analysis between SCFAs and expression of mRNA related to lipid metabolism in the liver. The values in the figure shows the correlation coeffcient. Blue circles show negative correlation and red circles show positive correlation. ACOX, Acyl-CoA oxidase; CPT1, Carnitine palmitoyl Table 1. CYP7a1, Cholesterol 7alpha-hydroxylase 1; DGAT1, Diacylglycerol acyltransferase-1; DGAT2, Diacylglycerol acyltransferase-2; FAS, Fatty acid synthase; GPAT, glycerol-3-phosphate acyltransferase; HMGCoAr, Hydroxymethylglutaryl-CoA reductase, reductase; LXR, Liver X receptor; SCD-1, Stearoyl-CoA desaturase 1; total SCFAs, total short-chain fatty acids.

Figure 7 shows the correlation coefficients by Spearman’s rank correlation analysis between the concentration of SCFAs in the cecum and mRNA expression in the liver. The concentration of acetic acid in the cecum negatively correlated with mRNA expression of ACOX, HMG-CoA reductase, and CYP7a1 in the liver (*p* < 0.05). The concentration of propionic acid in the cecum negatively correlated with mRNA expression of ACOX, CPT1, DGAT1, DGAT2, HMG-CoA reductase, and CYP7a1 (*p* < 0.05). The concentration of lactic acid in the cecum was negatively correlated with mRNA expression of CYP7a1 (*p* < 0.05). The concentration of succinic acid in the cecum negatively correlated with mRNA expression of CYP7a1 (*p* < 0.05). There was no relationship between the concentration of butyric acid in cecum and mRNA expression in the liver.

**Figure 8:**
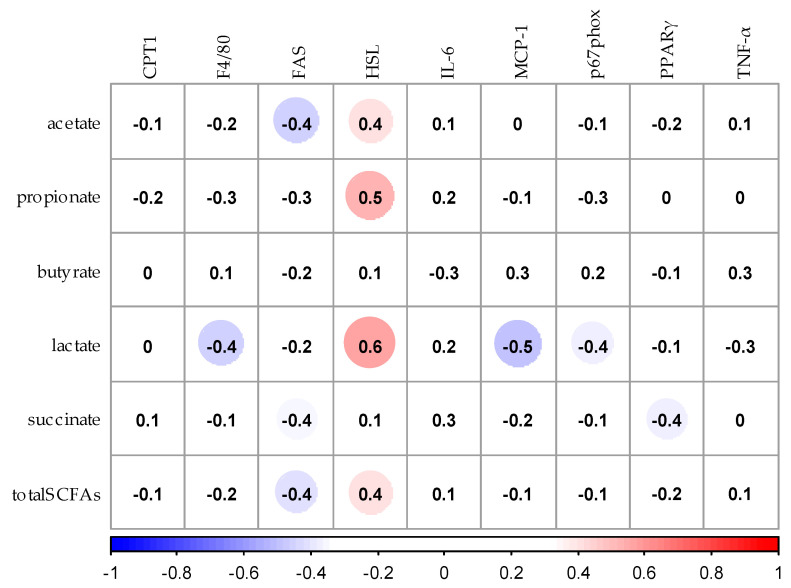
Spearman’s rank correlation analysis between SCFAs and expression of mRNA related to lipid metabolism in adipose tissues. The values in the figure shows the correlation coeffcient. Blue circles show negative correlation and red circles show positive correlation. CPT1, Carnitine palmitoyl transferase 1; FAS, Fatty acid synthase; HSL, Hormone-Sensitive Lipase; IL6, Interleukin-6; MCP-1, Monocyte chemotactic protein-1; p67phox, NADPH oxidase subunit p67 phox; PPARγ, Peroxisome proliferator-activated receptor-γ; TNF-α, Tumor necrosis factor-α; total SCFAs, total short-chain fatty acids.

The relationship between mRNA expression in adipose tissue and the concentration of SCFAs in the cecum is showed in Figure 8. The concentrations of propionic acid and lactic acid in cecum positively correlated with mRNA expression of HSL in adipose tissue (*p* < 0.05). The concentration of lactic acid in the cecum negatively correlated with mRNA expression of MCP-1 (*p* < 0.05).

## 4. Discussion

We investigated whether β-glucan-rich barley flour affected glucose and lipid metabolism at the gene expression level in high-fat obesity model mice by comparing them with a control group. Firstly, we performed OGTTs and found that barley flour intake reduced postprandial blood glucose levels in BF mice compared with the control mice. Barley β-glucan is known to moderate the digestion and absorption of carbohydrates in the intestine, which results in suppressing excessive elevation of postprandial blood glucose. Previous studies have shown that long-term barley intake suppressed elevated blood glucose levels in mice [29]. Brockman et al. reported that consumption of high concentrations of barley β-glucan improved glucose control during glucose tolerance tests [30]. Our results were consistent with these previous studies and suggested that barley intake improves glucose tolerance. Moreover, this effect may be regulated by SCFAs. A previous study speculated that barley suppresses food intake and improves insulin sensitivity through SCFAs, which promote intestinal hormone secretion, such as glucagon-like peptide 1 (GLP-1) from intestinal enteroendocrine cells [31]. GLP-1 has the ability to decrease blood glucose levels by modulating glucose-dependent insulin secretion [32]. We confirmed that barley intake increased SCFAs in the cecum in this study (Table 4); however, further studies are needed to elucidate the effect of gut hormone secretion on glucose metabolism. DNA microarray analysis showed that there was only a slight change in the expression level of genes related to glucose metabolism in ileum, liver and adipose tissues after barley intake. Therefore, we speculated that barley flour intake reduced postprandial blood glucose levels and was mainly influenced by retardation of glucose absorption and secretion of intestinal hormones, but not through changes in the expression of genes related to glucose metabolism in ileum, liver, and adipose tissues.

Intake of a diet rich in β-glucan barley improved lipid metabolism in mice. DNA microarray analysis showed that the expression of 50% of the genes related to steroid biosynthesis (mmu00100) pathways were greatly downregulated in the BF group compared with the control group in liver and adipose tissues. In particular, the mRNA expression level of HMG-CoA reductase, the rate-limiting enzyme in cholesterol synthesis, was lower in the BF group compared with the control group in the liver. Xia et al. also reported that intake of whole-grain significantly lowered the expression of HMG-CoA reductase in the liver and plasma and liver lipid levels when compared with the no barley group in high-fat diet model rats [33]. These reports are similar to the results obtained in our study. We found that intake of barley flour lowered liver and serum TC concentrations by consistently reducing the expression of genes involved in cholesterol synthesis. Moreover, it was previously suggested that improvements in lipid metabolism after barley intake is due to the promotion of cholesterol excretion in the intestine and inhibition of bile acid re-absorption. It is known that CYP7a1 is the rate-limiting enzyme for bile acid synthesis in the liver. A previous study reported that intake of barley β-glucan and waxy barley promotes excretion of bile acids in the feces; concomitantly increasing CYP7a1 expression and bile acid synthesis, thereby suppressing cholesterol synthesis [34]. However, in our study, the mRNA expression levels of CYP7a1 were significantly lower in the BF group compared with the control group. We also found that the fecal lipid levels did not change between the experimental and control groups (data not shown). The differing results may be due to differences in the source of barley, the experimental period and/or the animal model. Further studies are needed to elucidate how changes in the intake of barley affects bile acid metabolism.

The metabolic syndrome indices (abdominal fat organs, liver lipids, serum TC and LDL) is improved in mice fed barley flour: we previously reported that barley intake reduced fat accumulation in mice [35] and Choi et al. reported that consumption of barley β-glucan extract is involved in preventing obesity in mice fed a high-fat diet [36]. Another study reported that barley intake has an improving effect, both histologically and biochemically, on the liver of diabetic-model rats [37]. Similar effects have been shown in human studies; Japanese patients with hyperlipidemia had decreased abdominal fat and serum LDL after barley intake [38] and abdominal fat was decreased by whole-grain cereal intake in healthy humans [39]. Our results support previous studies. A DNA microarray showed that more than 10% of genes involved in glycerolipid metabolism (mmu00561) and the fatty acid elongation pathway (mmu00062) were downregulated in the BF group compared with the control group in the liver. q-PCR analyses also showed the expression of genes involved in lipid synthesis, such as DGAT1, SCD-1, FAS, and degradation, ACOX, CPT-1, were lowered after β-glucan-rich barley flour intake. The data suggest that gene expression levels related to lipid metabolism were lower overall in the liver, and affected an improvement in the obesity index.

Intake of β-glucan-rich barley flour affected the expression levels of mRNA related to lipid metabolism in adipocytes in a different way to the control group. Adipocytes are not only energy storage organs but also secrete some adipocytokines to regulate energy metabolism. In the present study, intake of barley flour downregulated genes related to several lipid metabolism pathways, such as steroid biosynthesis (mmu00100), and glycerolipid metabolism (mmu00561), in adipose tissue. It is considered that lipids flowing into adipose tissue were lower in the BF group compared with the control group due to lower lipid synthesis in the liver. The mRNA expression levels of HSL were significantly higher in the BF group compared with the control group. It is known that HSL catalyzes the degradation of triacylglycerol and decreases in the expression of HSL are associated with insulin concentration and obesity [40,41]. In this study, serum insulin concentration was reduced by barley flour intake (BF: 3.9 ± 1.5 ng/mL, control 6.0 ± 3.3 ng/mL); therefore, it was suggested that increased HSL expression occurs by the lower concentration of insulin accelerating fat degradation. Positive correlations between several SCFAs and HSL expression in adipose tissue were detected; however, further studies are needed to evaluate the relationship between HSL and SCFAs.

The expression levels of genes related to glucose and lipid metabolism were unchanged in the ileum. A previous study using fructo-oligosaccharides reported that succinic acid induced intestinal gluconeogenesis in vivo [42]; however, our study produced different results and this may be due to the differences in soluble fiber material. Therefore, our results suggest that the main organs in which barley flour is involved in lipid metabolism at the gene expression level are the liver and adipose tissues, but not the ileum.

Some inflammatory markers (MCP-1, p67^phox^, F4/80) were significantly decreased in the adipose tissue of barley-supplemented mice. It has been reported that adipocytes induce early inflammation by producing active oxygen via activation of NADPH oxidase in mice on a high-fat diet [43]. These results suggest that a decrease in adipocyte hypertrophy reduces fat synthesis in the liver, thus lowering visceral fat weight and inflammation in adipocytes.

Previous studies have described SCFAs as energy sources for the intestine and liver [44,45]. We found that several SCFAs negatively correlated with the expression levels of mRNA involved in hepatic lipid synthesis and degradation. Our results are supported by previous studies in which SCFAs suppressed lipid synthesis in the liver [46] and the expression levels of FAS and carbohydrate-response element-binding protein (Chrebp) were lower in mice fed SCFAs compared with mice fed cellulose [47]. Based on these results, we suggest that SCFAs alter the expression of genes related to lipid metabolism in the cecum. A recent study revealed that SCFAs influence the metabolism of lipids and glucose to regulate GLP-1 and transcription factor activity involved in lipid metabolism via intestinal SCFA receptors [48]. It is known that G protein-coupled receptors (GPCRs) are activated by SCFAs; notably, GPR43 and GPR41 regulate gut hormone secretion [49]. Further studies using mice deficient in these SCFA receptors will clarify the relationship between barley intake, SCFAs and the expression of genes related to lipid metabolism.

## 5. Conclusions

Our study provides evidence that β-glucan-rich barley flour affects lipid metabolism in the liver and adipose tissues at the gene expression level in high-fat obesity model mice. Using a DNA microarray, we observed that the intake of barley flour consistently reduced the expression levels of genes related to steroid biosynthesis in the liver and adipose tissue; however, in the ileum, barley flour is suggested to affect lipid metabolism through digestive absorption and intestinal fermentation, rather than changes in gene expression. The expression of genes involved in glucose metabolism did not change in each organ; therefore, we suggest that the improving effect of barley flour on postprandial blood glucose levels is caused by the modification of the gut events such as the retardation of glucose absorption and intestinal fermentation. In conclusion, microbiota-derived SCFAs affect changes in the expression levels of genes related to lipid metabolism in the liver and adipose tissues, thus affecting lipid accumulation and abdominal fat.

## Figures and Tables

**Table 1:** Nutritional components of Beau fiber (BF).

Beau Fiber (BF)	(g/100 g)
Moisture	8.1
Fat	3.0
Protein	8.8
Ash	0.1
Available carbohydrate	63.5
Total dietary fiber	16.5
β-Glucan	8.0

Available carbohydrate: (100 − (“Moisture” + ”Fat” + ”Protein” + ”Ash” + ”Total dietary fiber”)).

**Table 2:** Composition of the control and BF experimental diets (g/kg).

	Control	BF
Dextrinized corn starch	329.5	114.8
Casein	200.0	200.0
Sucrose	100.0	100.0
Soybean oil	70.0	70.0
Lard	200.0	190.9
Cellulose powder	50.0	-
BF *		303.8
AIN-93G mineral mixture	35.0	35.0
AIN-93 vitamin mixture	10.0	10.0
L-Cystine	3.0	3.0
Choline bitartrate	2.5	2.5
*t*-Butylhydroquinone	0.014	0.014
The amounts of β-glucan (%)	0.0	2.4

* BF obtained from the National Agriculture and Food Research Organization (Tsukuba, Japan).

**Table 3:** Final weight, body weight gain, food intake, and food efficiency ratio in mice fed the control and BF diets.

	Control	BF	*p* Value
Initial weight (g)	20.3 ± 0.9	20.3 ± 0.8	N.S.
Final weight (g)	41.5 ± 2.8	38.5 ± 4.0	N.S.
Body weight gain (g/day)	0.23 ± 0.03	0.20 ± 0.04	N.S.
Food intake (g/day)	3.3 ± 0.2	3.2 ± 0.1	N.S.
Food intake (g/day/10 g final weight)	0.79 ± 0.04	0.82 ± 0.05	N.S.
Food efficiency ratio (%)	7.0 ± 0.7	6.3 ± 1.0	N.S.

Values are mean ± SD, Food efficiency ratio (%) = Body weight gain/Food intake × 100, N.S.: not significant difference. BF: Beau fiber group.

**Table 4:** Organ weights in mice fed the control BF and diets.

	Control	BF	*p* Value
Liver (g)	1.50 ± 0.23	1.25 ± 0.11	*p* < 0.05
Epididymal fat (g)	2.45 ± 0.40	2.24 ± 0.54	N.S.
Retroperitoneal fat (g)	0.93 ± 0.15	0.74 ± 0.19	*p* < 0.05
Mesenteric fat (g)	0.95 ± 0.26	0.67 ± 0.18	*p* < 0.05
Cecum with digesta (g)	0.23 ± 0.05	0.42 ± 0.07	*p* < 0.05

Values are mean ± SD, N.S.: not significant difference.

**Table 5:** Concentrations of liver and serum lipids and hormones in mice fed the control and BF diets.

	Control	BF	*p* Value
*Concentration of liver lipids*			
Cholesterol (mmol/liver)	0.7 ± 0.1	0.4 ± 0.1	*p* < 0.05
(mmol/g liver)	0.5 ± 0.1	0.3 ± 0.1	*p* < 0.05
Triglyceride (mmol/liver)	8.0 ± 4.3	3.8 ± 1.5	*p* < 0.05
(mmol/g liver)	5.1 ± 1.9	3.0 ± 1.0	*p* < 0.05
*Biochemical levels in serum*			
Total cholesterol (mmol/L)	10.6 ± 0.7	8.8 ± 1.2	*p* < 0.05
LDL-cholesterol (mmol/L)	0.5 ± 0.1	0.4 ± 0.1	*p* < 0.05
HDL-cholesterol (mmol/L)	4.7 ± 0.2	4.6 ± 0.4	N.S.
Non-esterified fatty acid (NEFA) (μEq/L)	661.9 ± 63.7	666.5 ± 44.3	N.S.
Triglyceride (mmol/L)	2.4 ± 0.4	3.3 ± 0.8	*p* < 0.05
Insulin (ng/mL)	6.0 ± 3.3	3.9 ± 1.5	N.S.
Leptin (ng/mL)	92.8 ± 19.5	54.9 ± 22.1	*p* < 0.05

Values are mean ± SD, N.S.: not significant difference.

**Table 6:** Concentration of short-chain fatty acids (SCFAs) and organic acids in cecum contents.

(μmol/cecum)	Control	BF	*p* Value
Acetic acid	2.2 ± 0.9	3.7 ± 1.0	*p* < 0.05
Propionic acid	0.7 ± 0.3	1.4 ± 0.4	*p* < 0.05
Butyric acid	0.7 ± 0.2	0.9 ± 0.5	N.S.
Lactic acid	0.2 ± 0.0	0.4 ± 0.1	*p* < 0.05
Succinic acid	0.1 ± 0.1	0.3 ± 0.1	*p* < 0.05
Total short-chain fatty acids (SCFAs)	4.5 ± 1.5	7.7 ± 2.0	*p* < 0.05

Values are mean ± SD, N.S.: not significant difference.

## Supplementary Materials

The following are available online at https://www.mdpi.com/2072-6643/12/11/3546/s1, Table S1: Primers used in the real-time reverse transcription polymerase chain reaction. Table S2: Differentially expressed genes in the ileum by microarray analysis. Table S3: Differentially expressed genes in the liver by microarray analysis., Table S4: Differentially expressed genes in the adipose tissue by microarray analysis.
